# Post-Traumatic Giant Left Ventricular Pseudoaneurysm: a Multimedia Presentation

**DOI:** 10.21470/1678-9741-2019-0169

**Published:** 2020

**Authors:** Danilo Tadao Wada, Marcel Koenigkam-Santos, André Schmidt, Alfredo José Rodrigues, Paulo Roberto B. Evora

**Affiliations:** 1 Centro de Ciências das Imagens e Física Médica, Hospital das Clínicas da Faculdade de Medicina de Ribeirão Preto - Universidade de São Paulo (HCFMRP-USP), Ribeirão Preto, SP, Brazil.; 2 Faculdade de Medicina de Ribeirão Preto - Universidade de São Paulo (FMRP-USP), Ribeirão Preto, SP, Brazil.

**Keywords:** Aneurysm, False, Wounds, Stab, Heart Ventricules, Myocardium, Sutures

## Abstract

Traumatic left ventricular pseudoaneurysms are rare and surgical correction is the treatment of choice. In this article, it is reported a case of a myocardial stab injury with primary suture and development of a giant pseudoaneurysm, five years later, that underwent surgical repair.

**Table:** 

Abbreviations, acronyms & symbols	
CPB	= Cardiopulmonary bypass
LV	= Left ventricle
LVP	= Left ventricular pseudoaneurysm
MRI	= Magnetic resonance imaging

## INTRODUCTION

Left ventricular pseudoaneurysms (LVP) form in the presence of a cardiac rupture contained by surrounding structures, like the pericardium or scar tissue^[1]^. Thus, unlikely true aneurysms, a LVP contains no endocardium or myocardium, being more propense to ruptures^[2-4]^. Trauma is a rare cause, given the high mortality in this scenario^[5]^.

Because patients frequently present with nonspecific symptoms, a high index of suspicion is needed to make the diagnosis. Whereas electrocardiography and chest X-ray abnormalities are almost always present, they are also usually nonspecific^[1]^.

This is the report of the investigation and treatment of a case of penetrating thoracic trauma by stab with initial surgical repair evolving to a giant LVP after five years of indolent evolution.

## PATIENT CHARACTERIZATION

### Clinical Data

A 34-year-old female, inmate, reported intermittent episodes of exercise-related chest pain and mild dyspnea. The patient reported a traumatic stab injury in the chest five years before that underwent surgery with suture of pulmonary and myocardial lacerations.

In the physical examination, patient had blood pressure and heart rate between normal values with a regular two-stroke heart rhythm. Continuous 4+/6+ murmur with fremitus and cervical irradiation were noted.

### Radiography

**Fig. 1:**
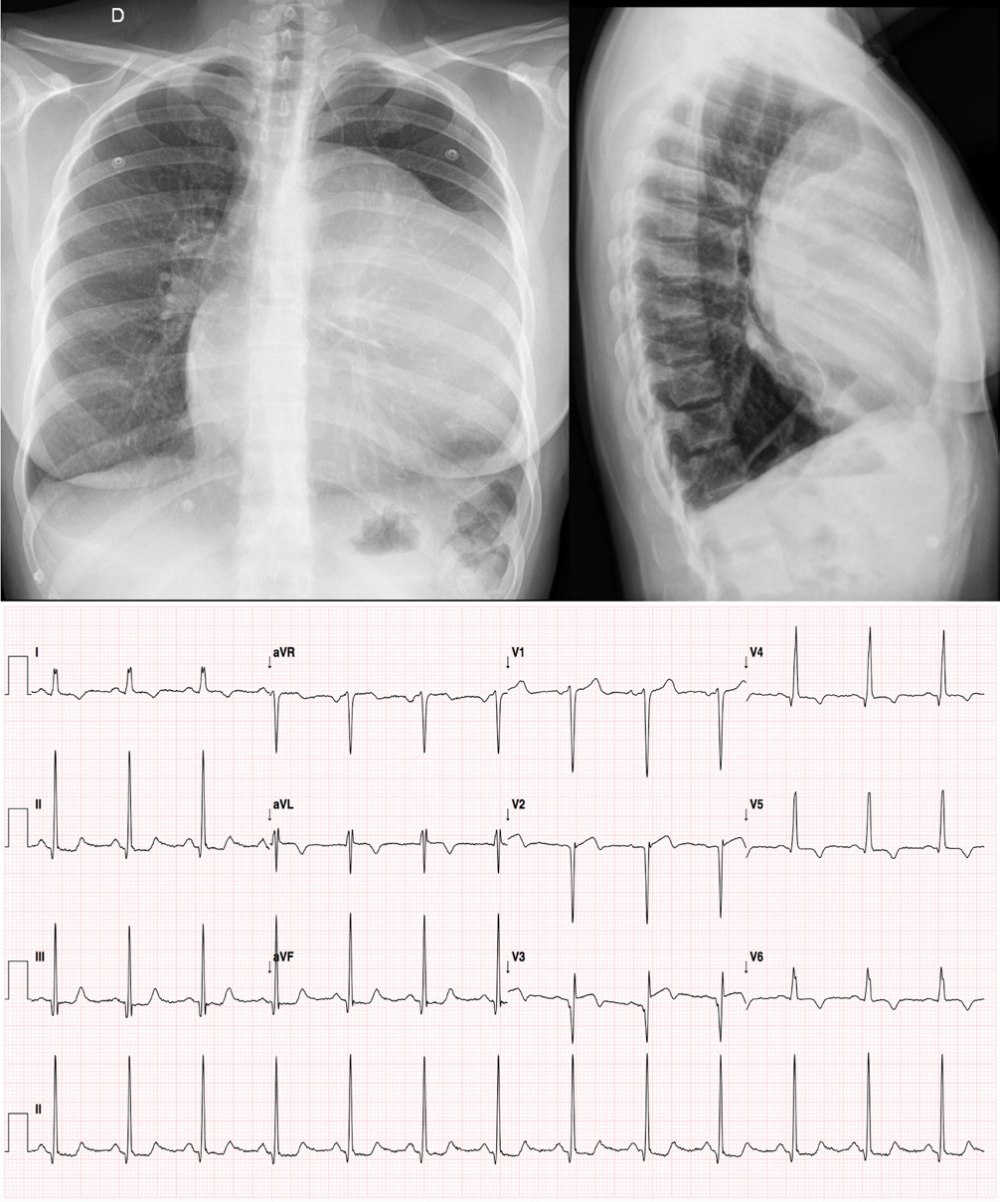
Frontal (upper left) and profile (upper right) chest radiographies and electrocardiography (bottom) showing sinusal rhythm with signs of left ventricular overload.

Mediastinal mass with left cardiac silhouette lost and opacification of the retrosternal space is shown (Figure 1).

### Electrocardiography

Sinusal rhythm with signs of left ventricular overload (Figure 1).

### Transthoracic Echocardiography with Doppler Evaluation

Moderate dilatation of left ventricle (LV) with a large pseudoaneurysm in the mid-anterior wall. Severe ejection fraction impairment (31%) with mid-anterior and apical anterior hypokinesis (Video 1). Bidirectional flow is seen between the LV and the pseudoaneurysm cavity (Video 2).

**Video 1:**
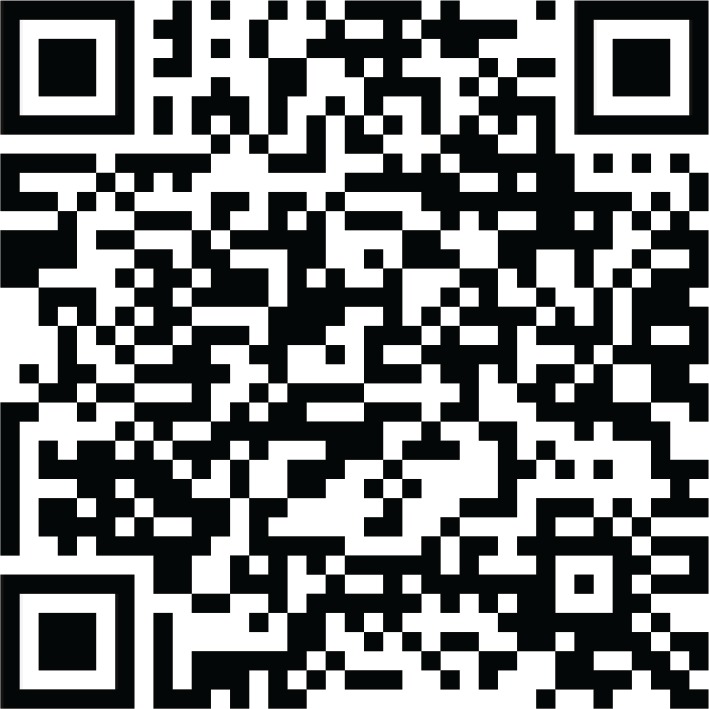
Transthoracic echocardiography: left ventricular pseudoaneurysm neck and anterior wall hypokinesis.

**Video 2:**
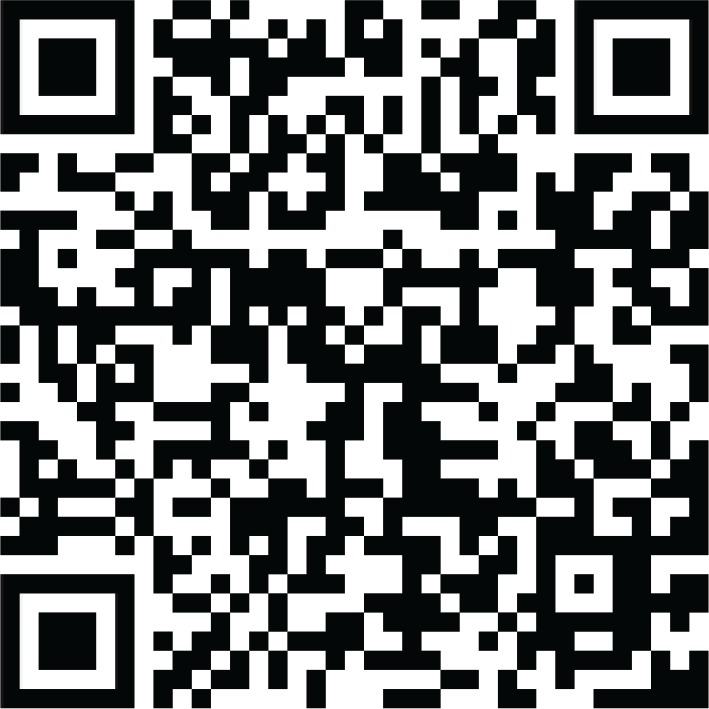
Transthoracic echocardiography, Doppler evaluation: bidirectional flow between the left ventricle and the left ventricular pseudoaneurysm cavity.

### Cardiac Magnetic Resonance Imaging

**Fig. 2:**
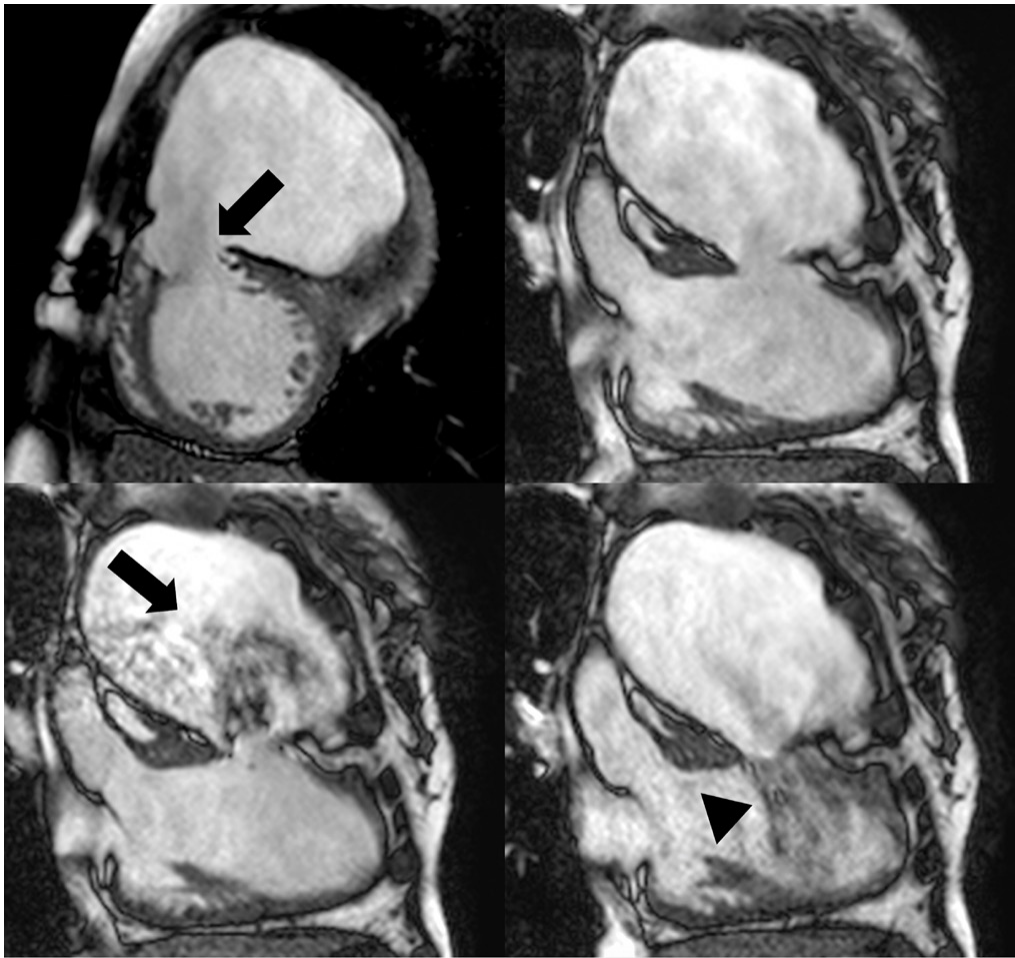
Left ventricular short-axis cinemagnetic resonance image (upper left) showing the mid-anterior wall disruption point (arrow). Oblique left ventricular long-axis cine images during end diastole (upper right), systole (bottom left), and mid diastole (bottom right). Turbulent flow is depicted from the left ventricle to the pseudoaneurysm in the systole (arrow), with reflow during diastole (arrowhead).

Magnetic resonance imaging (MRI) was able to precisely evaluate the focal disruption of the left ventricular mid-anterior wall (Figure 2 and Video 3), also allowing visualization of the entire LVP, helping to exclude thrombus in it. Signs of myocardial ischemic disease were also depicted (not shown in the figures).

**Video 3:**
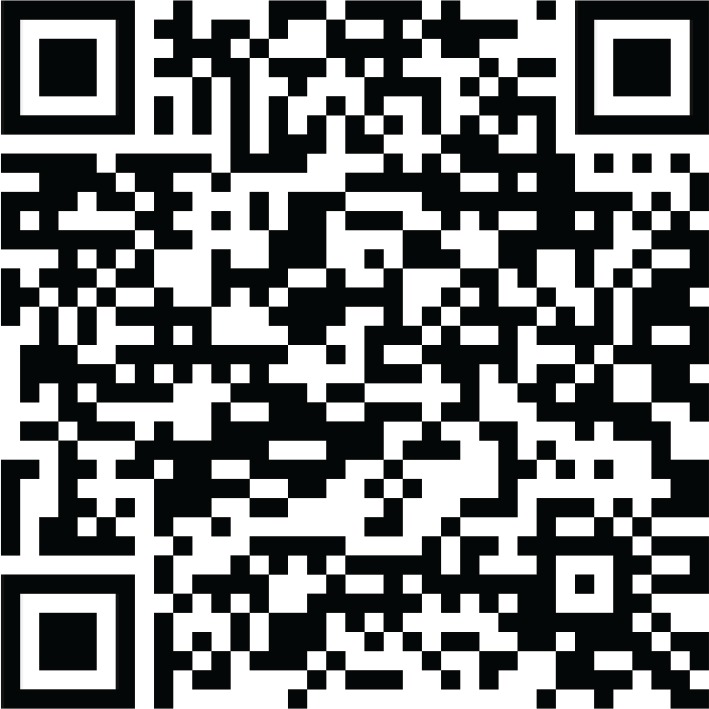
Magnetic resonance imaging, left ventricular shortaxis cine imaging: left ventricular anterior wall disruption and left ventricular pseudoaneurysm.

### Cardiac Catheterism

**Fig. 3:**
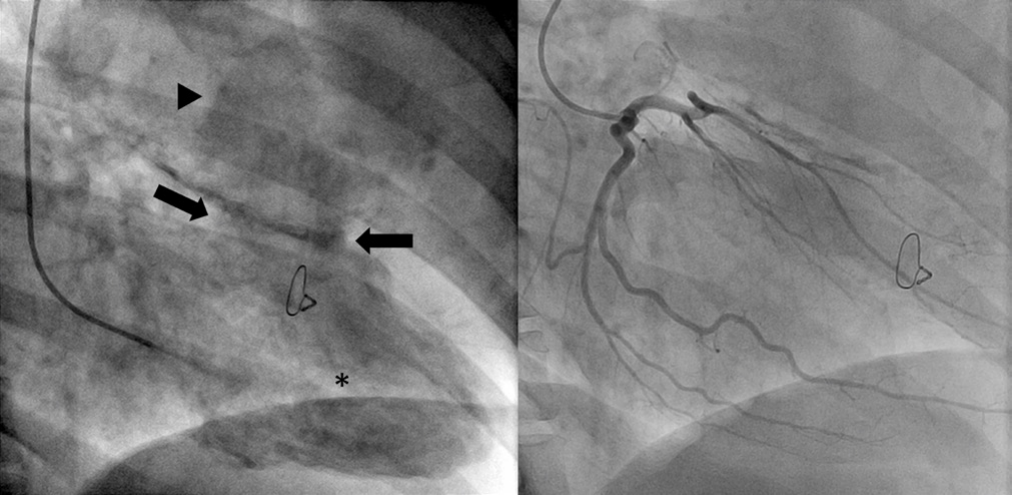
Cardiac ventriculography (left) showing the neck of the pseudoaneurysm (arrows) in the left ventricular (*) anterior wall with turbulent flow in its cavity (arrowhead). Left coronary catheterism is also shown (right).

Large pseudoaneurysm cavity with complete obstruction of mid segment of left anterior descending coronary artery at the level of LVP neck (Figure 3, Video 4, and Video 5).

**Video 4:**
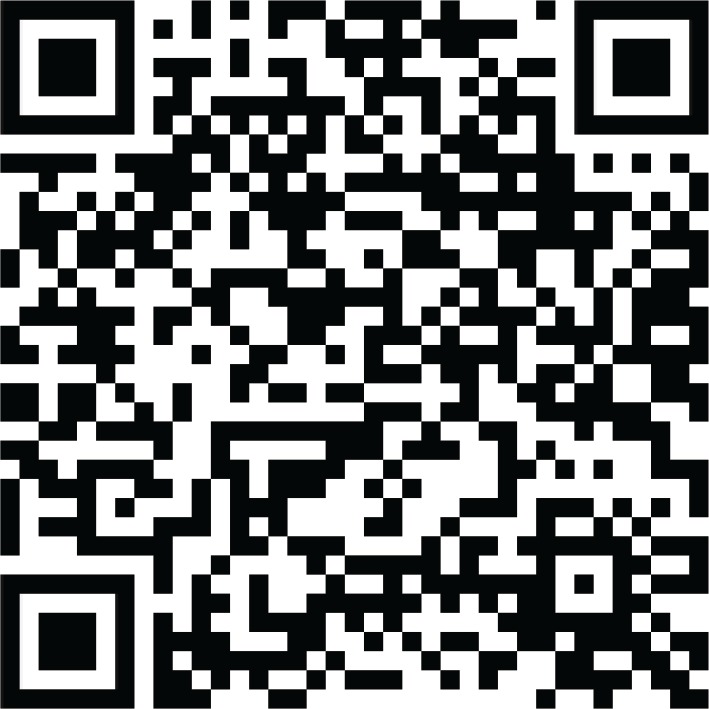
Magnetic resonance imaging, left ventricular long-axis cine imaging: bidirectional turbulent flow.

**Video 5:**
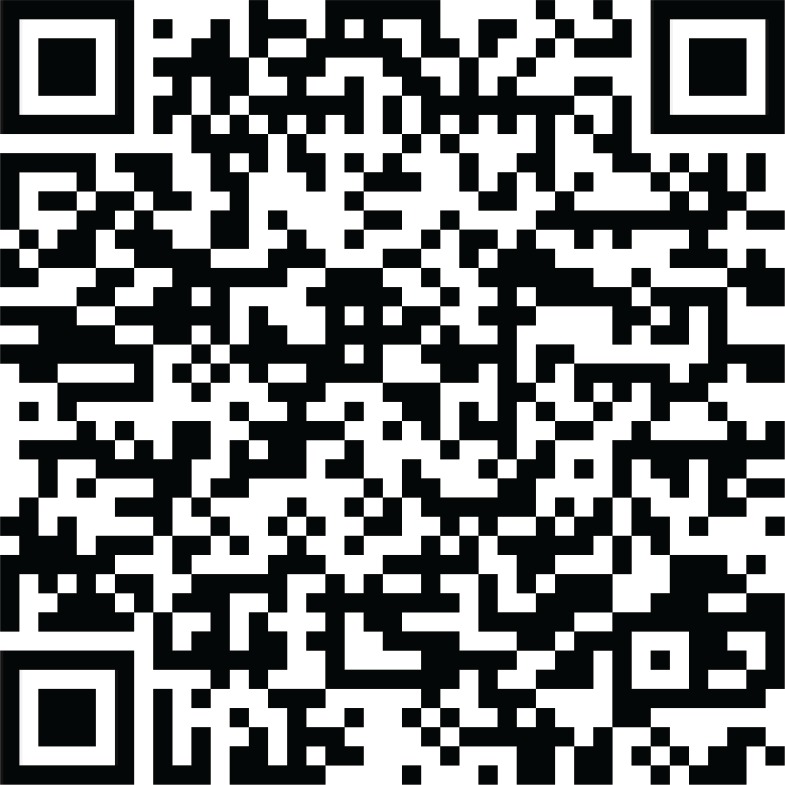
Cardiac ventriculography: left ventricle and left ventricular pseudoaneurysm.

## DESCRIPTION OF THE TECHNIQUE EMPLOYED

The patient underwent surgical correction of the pseudoaneurysm by direct suture with good evolution. Medium sternotomy was performed after femoro-femoral cardiopulmonary bypass (CPB) had been established. The patient was cooled down to 28ºC. The ascending aorta was not cross-clamped. The fibrous wall of the pseudoaneurysm was identified and its medial surface (contiguous to the anterior mediastinum) was widely opened during a short period of circulatory arrest and ventricular fibrillation, in order to depressurize the LV and the pseudoaneurysm and to prevent air embolism. The left ventricular wound was identified (3.5 cm ´ 5 cm) and the CPB restarted. The wound was closed using a bovine pericardial patch sutured to its margin with horizontal mattress stiches (Ethibond 2.0, Ethicon®) during ventricular fibrillation. Maneuvers to remove air from the LV were performed before tying the last stich. Besides a mediastinal drain, a suction drain was positioned inside of the pseudoaneurysm cavity (left open to the anterior mediastinum) and the chest was closed as usual. The suction drain was removed few days later, when the serosanguineous drainage was < 100 ml/24 h.

### Clinical Follow-up

Another cardiac MRI was made six months latter showing a minimal residual cavity without signs of complication. After surgery, the patient had a good postoperative evolution without complications, being discharged from hospital after 11 days. She is at clinical and imaging follow-up, with a two-year evolution, without worsening of symptoms.

## DISCUSSION

The incidence of LVP is low and most of the cases are related to acute myocardial infarction, cardiac surgery, and trauma. LVP have become a rare complication of acute myocardial infarction, occurring in approximately 2% of the cases or less, when thrombolytic or primary percutaneous intervention can be performed^[6,7,8]^.

The natural history of surgically treated and untreated LVP is not clearly defined^[9]^ and data to guide the treatment are scarce^[10]^. Surgical repair is the recommended treatment, given the pseudoaneurysms’ propensity for life-threatening complications, such as cardiac tamponade and shock. Untreated pseudoaneurysms have a 30% to 45% risk of rupture and the surgical repair of post-traumatic LVP has a reported mortality of > 7%.

Is this article, it is reported an unusual case of post-traumatic giant LVP with an indolent evolution, although all the potential risks of rupture.

**Table:** 

Authors' roles & responsibilities	
DTW	Drafting the work or revising it critically for important intellectual content; final approval of the version to be published
MKS	Drafting the work or revising it critically for important intellectual content; final approval of the version to be published
AS	Drafting the work or revising it critically for important intellectual content; final approval of the version to be published
AJR	Drafting the work or revising it critically for important intellectual content; final approval of the version to be published
PRBE	Substantial contributions to the conception or design of the work; drafting the work or revising it critically for important intellectual content; final approval of the version to be published
